# 

**Published:** 1888-12

**Authors:** 


### INDEX TO VOLUME LVII.

### A

PAGE.
Abdominal and Vaginal Section, Twelve Months of. Byford.................... 257
Announcements:
Allegheny County Medical Society...... 352
American Academy of Medicine.......... 320
American Public Health Association..320, 356
Congress of American Physicians and Surgeons............................ 191
International Medical Congress of Australia 256
Sanitary Convention at Hastings, Michigan, December 3 and 4, 1888.......... 256
American Ophthalmological Society...... 64
American Otological Society ............ 64
American Rhinological Society.......... 64
Alumnæ Association of the Woman's Medical College of the New York Infirmary, Annual meeting, 1888.................. 147
American Academy of Medicine........... 349
American Association of Obstetricians and Gynæcologists......................... 279
American Gynæcological Society........... 230
American Ophthalmological Society ...103, 234
American Otological Society ...........101, 232
American Orthopedic Association.......... 235
American Physicans and Surgeons, Congress of............................ 205
American Rhinological Association......... 240
American Surgical Association.... ....... 219
Ampullary Dilatations, The Value of....... 186
Antipyrin, Hypodermic Injections of....... 320
Antipyrin in Polyuria and Diabetes........ 59
Antipyrin, Use of, with Quinine........... 320
Association of American Physicians....... 213
Astigmatism, Binocular. S. O. Richey..... 4

### B

Behrens, B. M. Letter.................... 255
Berlin Medical Society.................... 113
Bologna, The Festival of the Eight Hundreth Anniversary of the University of... 115
Book Reviews and Notices .62, 127, 186, 314, 376
Books Received.....................188, 377
PAGE
British Medical Association............64, 150
Bronchitis, Formula for................... 320
Bromine as a Disinfectant for Streets....... 313
Burns, Treatment of...................... 186
Byford, H. T. Twelve Months of Abdominal and Vaginal Section................ 257

### C

Caldwell, W. C. Pathology and Treatment of Dry Tongue.......................... 268
Canadian Medical Association............. 313
Cascara Sagrada in Rheumatism........... 184
Casselberry, W. E. A New Method of Feeding in Intubation of the Larynx......... 201
Census, The United States................ 312
Chicago Medical Society:
Regular Meeting, May 7............ .... 137
Regular Meeting, May 21................ 139
Regular Meeting, June 4................. 141
Regular Meeting, July 2................. 243
Chicago Pathological Society:
Regular Meeting, June.................. 11
Regular Meeting, August................ 143
Regular Meeting, October............... 306
Regular Meeting, November.............. 335
Children, What Kills the.................. 178
Chorea of Childhood, The Eyes in.......... 58
Congenital Hernia and Hydrocele.......... 185
Congress of American Physicians and Surgeons.................................. 205
Conheim, Monument to.................... 64
Correspondence, Domestic. Behrens....... 255

### D

Danforth, I. N. Four Cases of Surgical Kidney................... ................ 74
Diabetes, Lithiated Pills for.............. 64
Diabetes, Seminolina in.................. 313

PAGE
Diaphoresis, Value of, in Infectious Diseases. 384
Dry Tongue, Pathology and Treatment of. Caldwell ........................ 268
Dublin Medical Schools, The Amalgamation of........................ 184

E

Edinburg, Medico-Chirurgical Society of..43, 106
Erysipelas, Treatment of Medical........... 64
Extracts and Abstracts........................ ............54, 62, 121, 127, 255, 310, 362

F

Foreign Correspondence. The Festival of Eight Hundredth Anniversary of the University of Bologna. Lagorio, A...... 115
Italian Medical Congress. Lagorio, A... 359

G

Gastrostomy. Holmes .................... 330
Grafting Skin of Fowls. Rogers, B. F.... 333
Gynæcological Society of Chicago:
Regular Meeting, April 20............... 13
Regular Meeting, May 25... ........... 86
Regular Meeting, June 29................ 248
Regular Meeting, September 28.......... 340
Annual Meeting, October 19............. 349

H

Hernia and Hydrocele, Congenital......... 185
Holmes, Bayard. Secondary Mixed Infection in Typhoid Fever..................... 65
Holmes, Bayard. The Destruction of the Trophic Function as an Element in Determining the Localization of Infection..... 128
Holmes, Bayard. Gastrostomy............ 330

I

Illinois Medical-Practice Act. Johnson, H. A. 227
Illinois State Board of Health, Abstract of Quarterly Meeting...................... 157
International Otological Congress.......... 242
Intubation of the Larynx, A New Method of Feeding in. Casselberry............... 201
Iodism, Fugitive.......................... 185

J

PAGE
Jaw, Subperiosteal Removal of Lower, &c.. 58
Johnson, H. A. Illinois Medical-Practice Act 327
Joint and Bone Tuberculosis. Van Hook.. 321

K

Kidney, Surgical, Four Cases of. Danforth 74

L

Lagorio, A. Italian Medical Congress...... 359
Lagorio, A. The Festival of the Eight Hundredth Anniversary of Bologna...... 115
Lanolin and its Imitations................. 183
Larynx, A Man Without a ................ 59
Larynx, A New Method of Feeding in Cases of Intubation of. Casselberry...... 201
Leprosy in Russia......................... 61
Liver, Some Practical Deductions Drawn from the Known Functions of the. Webster............................... 193
London, Clinical Society of............... 104

M

Malignant Pustule among Wool Sorters.... 180
Medical Profession, The Re-establishment of the. An address..................... 120
Membrana Tympani, The Primary Physiological Purpose of the. Richey.......... 197
Mental Overwork, Effects of .............. 181
Michigan State Board of Health .......... 99
Michigan State Medical Society ........... 39
Miscellany...........................64, 189, 319
Mississippi Valley Medical Association...... 300
Modern Medical Curriculum, The ......... 310

O

Obituary:
E. Williams............................ 279
H. D. Schmidt.......................... 361
H. B. Sands............................ 62

P

Pamphlets Received......................189, 378
Psoriasis, Wash for Syphilitic............. 64
Psychological Laboratories ................ 182

R

Rheumatism, Value of Cascara Sagrada in.. 184
Richey, S. O. Binocular Astigmatism..... 4
Richey, S. O. The Primary Physiological Function of the Membrana Tympani...... 197
Rogers, B. F. Grafting Skin of Fowls. ... 333

S

PAGE

Sandwich Islands, Danger from the........ 64
Sanitary Convention at Albion, Michigan... 157
Seminolina in Diabetes.................... 313
Skin Disease, Two Interesting Cases of. Wing. 1

T

Tarsectomy for Senile Ectropion........... 61
Tōkyō Medical Society, Recent Meeting of.. 319
Tongue, Pathology and Treatment of Dry. Caldwell ............................ 268
Tracheotomy, The Unsuitability of Silver Tubes for............................ 54
Tribromophenol as an Antiseptic........... 319
Trophic Function, Destruction of the, as an Element in Determining the Localization of Infection. Holmes ..................... 128
Tuberculosis, Recent Observations on...... 61
Tumors, On the Treatment of Sebaceous... 62
Typhoid Fever, Cardiac and Arterial Affections after.......................... 56
Typhoid Fever, Secondary Mixed infection in. Holmes.......................... 65

U

United States Census...................... 312
University Medical Magazine............... 320
Uric Acid, The Formation and Excretion of. 171
Uterus, Bacteriological Examination of, after Parturition........................... 60

V

Van Hook. Joint and Bone Tuberculosis.. 321
Virginia Examining Board and Medical Graduates.............................. 64

W

Webster, G. W. Some Practical Deductions Drawn from the Known Functions of the Liver ............................... 193
Williams, Elkanah, M. D., Obituary of......279

PAGE

Wing, Elbert. Two Interesting Cases of Skin Disease.......................... 1
Wisconsin State Medical Society........... 36

X

Xanthelasma of the Heart................. 64

Y

Yellow Fever, Investigations Relating to Etiology and Prophylaxis of............. 255
Yellow Fever, New Method of Treating... 176

### CONTRIBUTORS TO THIS VOLUME.

BYFORD, H. T.

CALDWELL, W. C.

CASSELBERRY, W. E.

DANFORTH, I. N.

HOLMES, BAYARD.

RICHEY, S. O.

ROGERS, B. F.

VAN HOOK, WELLER.

WEBSTER, G. W.

WING, ELBERT.

Editorial:
Cholera and Inoculation.................. 136
Contagious Affections.................... 85
Exercise and Fatty Heart................. 277
Higher Medical Education................. 83
Kolpo-Uretero-Cystotomy ................. 6
New Antipyretics and Proprietary Medicines.................................. 84
Ptomaïnes and their Effects ............. 334
Surgery of the Brain..................... 203
The Congress of American Physicians and Surgeons.............................. 204
The Late Emperor of Germany.......... 10
The Need of a Medical Reference Library. 278
The New Quarantine Law.................. 134
The Outbreak of Yellow Fever........... 204
The Paris Congress for the study of Tuberculosis................................ 135
Useful Memorials......................... 335
Yellow Fever............................. 137

### BOOKS RECEIVED.

Chemical Experiments for Medical Students. By W. S. Christopher, M. D.

Transactions of the Thirty-eighth Annual Meeting of the Illinois State Medical Society. 1888.

The Physicians' Interpreter. In Four Languages. By F. A. Davis.

Index Catalogue of the Library of the Surgeon-General's Office, U. S. Army. Vol. IX.

Ptomaïnes and Leucomaïnes. By Victor C. Vaughan, Ph. D., M. D., and F. G. Nooy, M. S.

Manual of Chemistry. A Guide to Lectures and Laboratory Work, etc. By W. Simon, Ph. D., M. D.

A Text-Book of Pharmacology, Therapeutics, and Materia Medica. By T. Lauder Brunton, M. D., D. Sc., F. R. S.

A Manual of General Pathology. By Joseph F. Payne, M. D. Oxon., F. R. C. P.

Therapeutics; Its Principles and Practice. Seventh edition. By H. C. Wood, M. D., LL. D.

A System of Gynæcology. By American Authors. Vol. II.

Hand-Book of Historical and Geographical Phthisiology. By George A. Evans, M. D.

Fifteenth Annual Report of the Secretary of the State Board of Health of Michigan. 1887.

A Manual of Ophthalmic Practice. By Charles Higgens, F. R. C. S. E.

The Urine and the Common Poisons. By J. W. Holland, M. D.

The Diseases of the Chest. By Vincent D. Harris, M. D., F. R. C. P.

Practical Anatomy. Seventh edition. By Christopher Heath, F. R. C. S.

The Ear and its Diseases, etc. By Samuel Sexton, M. D.

Landmarks, Medical and Surgical. By Luther Holden.

A Text-Book of Human Physiology. By Austin Flint, A. M., M. D.

Treatise on the Diseases of Women. By Alexander J. C. Skene, M. D.

Second Annual Report of the State Board of Health of the State of Pennsylvania. 1886.

A Manual of Dietetics. By W. B. Pritchard, M. D.

Morrow's Atlas of Venereal and Skin Diseases. Fasciculi 7 and 9.

### PAMPHLETS RECEIVED.

Cellular Digestion. Its Utility in Pathological Processes. By N. S. Davis, Jr., A. M., M. D.

Conditions Rendering Diagnosis Difficult in Pelvic and Abdominal Diseases. By T. B. Harvey, M. D. LL. D.

The American Hip-Splint. By A. B. Judson, M. D.

Annual Report of the Commissioner of Pensions for 1888.

Transactions of the Medical Association of the State of Missouri for 1888.

The Orthopedic Treatment of Paralysis of the Anterior Muscles of the Thigh. By A. B. Judson, M. D.

Transactions of the Medical Society of the State of West Virginia.

Surgery as a Science and an Art. By George E. Frothingham, M. D.

On the Detection of Coloring Matters in Whisky. By J. H. Long.

On the Stability of the Thallons Iodide, and the Determination of Thallium. By J. H. Long.

On the Densities and Refraction Indices of Certain Oils. By J. H. Long.

A Synopsis of the Medical Botany of the United States. By J. M. G. Carter, A. M., M. D., Ph. D.

The Medical News Visiting List for 1889. Lea Brothers & Company.

The Physicians' Visiting List for 1889. P. Blakiston, Son & Company.

Uterine Fibroids Treated by Ergot. By Daniel T. Nelson, A. M., M. D.

Report on Hydrophobia. By Charles W. Dulles, M. D.

The Causation of Cold Weather Diseases. By Henry B. Baker, M. D.

On Bacterial Invasion of the Inner Ear in the Course of Diphtheria. By S. Moos, Heidelberg. Illustrated by H. S. B. McCauley, M. D.

The Preferable Climate for Phthisis. By Charles Denison, A. M., M. D.

The Operation for Stellate and Bilateral Laceration of the Cervix Uteri. By Hubbard W. Mitchell, M. D.

Recent Advances in State Medicine. By Henry B. Baker, M. D.

Carie Del Temporale. Pil. Dott. Emelio de Rossi.

Medical Communications of the Massachusetts Medical Society.

Experimental Researches Respecting the Relation of Dress to Pelvic Diseases. By J. H. Kellogg, M. D.

Inebriate Asylums and their Work. By T. D. Crothers, M. D.

The Primary Purpose of the Membrana Tympani. By S. O. Richey, M. D.

Nystagmus in Connection with Diseases of the Ear. By Charles J. Kipp, M. D.

The Contagiousness of Phthisis. By Lawrence F. Flick, M. D.

The Physicians' Pocket Day Book. Designed by C. H. Leonard, M. A., M. D.

Volume LVII.] DECEMBER, 1888. [Number 6.

### The CHICAGO MEDICAL JOURNAL AND EXAMINER

EDITOR:
S. J. JONES, M.D., LL.D.

COLLABORATORS:

CHICAGO:

Professor E. ANDREWS, M.D., LL.D.
Professor J. BARTLETT, M.D.
Professor W. T. BELFIELD, M.D.
Professor F. BILLINGS, M.D.
Professor W. H. BYFORD, A.M., M.D.
Professor L. CURTIS, A.M., M.D.
Professor N. S. DAVIS, Jr., A.M., M.D.
Professor O. C. DE WOLF, A.M., M.D.
Professor C. FENGER, M.D.
Professor J. N. HYDE, A.M., M.D.
Professor E. F. INGALS, A.M., M.D.
Professor F. S. JOHNSON, A.M., M.D.
Professor H. A. JOHNSON, M.D., LL.D.
Professor C. W. PURDY, M.D.
Professor C. G. SMITH, A.M., M.D.
Professor E. WING, A.M., M.D.
Professor E. C. DUDLEY, A.B., M.D.

MILWAUKEE, WIS.:
Professor N. SENN, M.D., Ph.D.

WASHINGTON, D. C.:
S. O. RICHEY, M. D.

UNITED STATES ARMY:
Surgeon D. L. HUNTINGTON, M.D.

UNITED STATES NAVY:
Surgeon-General J. M. BROWNE, M.D. Medical-Director A. L. GIHON, A.M., M.D.

MARINE HOSPITAL SERVICE:
Surgeon S. T. ARMSTRONG, M.D., Ph.D.

